# Bioinformatic tools for tRNA gene analyses in mitochondrial DNA sequence data

**DOI:** 10.1016/j.dib.2020.105284

**Published:** 2020-02-22

**Authors:** Elena V. Romanova, Yurij S. Bukin, Dmitry Yu. Sherbakov

**Affiliations:** a Laboratory of Molecular Systematics, Limnological Institute, Irkutsk, Russian Federation; b Faculty of Biology and Soil Studies, Irkutsk State University, Irkutsk, Russian Federation

**Keywords:** tRNA genes, Mitochondrial genomes, Sequence alignment, Genetic distance, R script

## Abstract

The data presented here are related to the research article entitled “Hidden cases of tRNA genes duplication and remolding in mitochondrial genomes of amphipods” (Romanova et al., 2020) [1]. Correct tRNA gene sequence annotation in mitochondrial (mt) and nuclear genomes sometimes can be a challenging task because of the differential performances of tRNA annotation/prediction programmes. These programmes may cause false positive or false negative predictions. Moreover, additional difficulties with annotation may be caused by the presence of duplicated tRNA genes and those coding tRNAs with altered identities occurring as due to a mutation in their anticodon sequence (tRNA gene remolding/recruitment).

We developed an R script automating the diagnosis of ancestor tRNA gene coding specificity regardless of anticodon sequence based on genetic distance comparison. Some of the predicted tRNA genes from the mt genomes of amphipods are presented. We also developed an R script for estimation of the best mode of sequence alignment, which was applied to determine the best alignment of tRNA genes in [1], but is also suitable for testing of any nucleotide alignment sets used in phylogenetic inferences.

**Table:** Specifications Table

Subject	Biochemistry, Genetics and Molecular Biology
Specific subject area	Bioinformatic studies of mitochondrial tRNA gene sequences
Type of data	Code, Table, Figure
How data were acquired	Codes, *In silico* analysis of tRNA gene sequences performed using R scripts.
Data format	Raw, R scripts text, analysed data
Parameters for data collection	tRNA gene sequences from available mitochondrial (mt) genomes of amphipods were predicted using MiTFi [2]
Description of data collection	Custom written R scripts. Analysis of tRNA gene sequences from amphipod mt genomes performed using R scripts.
Data source location	Limnological Institute, Irkutsk, Russia
Data accessibility	The raw data files are provided in the Data in Brief article. The text of codes and the examples of input and output files were deposited in a repository.
Related research article	Romanova, E.V., Bukin, Y.S., Mikhailov, K.V., Logacheva, M.D., Aleoshin, V.V., Sherbakov, D.Yu. 2020. Hidden cases of tRNA genes duplication and remolding in mitochondrial genomes of amphipods. *Molecular Phylogenetics and Evolution*, 144, 106710.

**Table:** 

**Value of the Data** • The data provide a useful tool for the selection of an optimum mode of alignment of any set of nucleotide sequences that is essential for robust phylogenetic inferences. • The data present a bioinformatics tool to define true tRNA gene identity regardless of its codon sequence. This will help with performing correct annotation of the tRNA genes in mt genomes and with identifying the true origination of tRNA gene copies that underwent changes to their identity through a mutation in their codon sequence (tRNA gene remolding). • The data from the pairwise identity analysis estimated for duplicated and some single tRNA genes of interest in mt genomes of amphipods provide additional evidence about the true evolutionary origin of these genes that is useful for understanding their evolutionary dynamics.

## 1 Data

The data describes the two R scripts: 1) The script for the identification of tRNA gene isoacceptor types based on genetic distance analysis. Additionally, the data from the genetic distance analysis of duplicated and some single tRNA genes from the mt genomes of amphipods are presented in Table S1. 2) The script for choosing the best mode of sequence alignments and the output of its application based on differently aligned groups of tRNA genes from the mt genomes of amphipods are presented in Table S2.

## 2 Experimental design, materials, and methods

### 2.1 R script for estimation of tested tRNA gene sequence resemblance to certain isoacceptor type

For the cases when it is necessary to verify tRNA gene sequence predictions by different programmes or to distinguish between original and remolded/recruited tRNA genes [3,4] we developed an R script that performs identification of tRNA isoacceptor type based on estimation and comparison of genetic distance data using “ape” package [5]:

**Figure:**
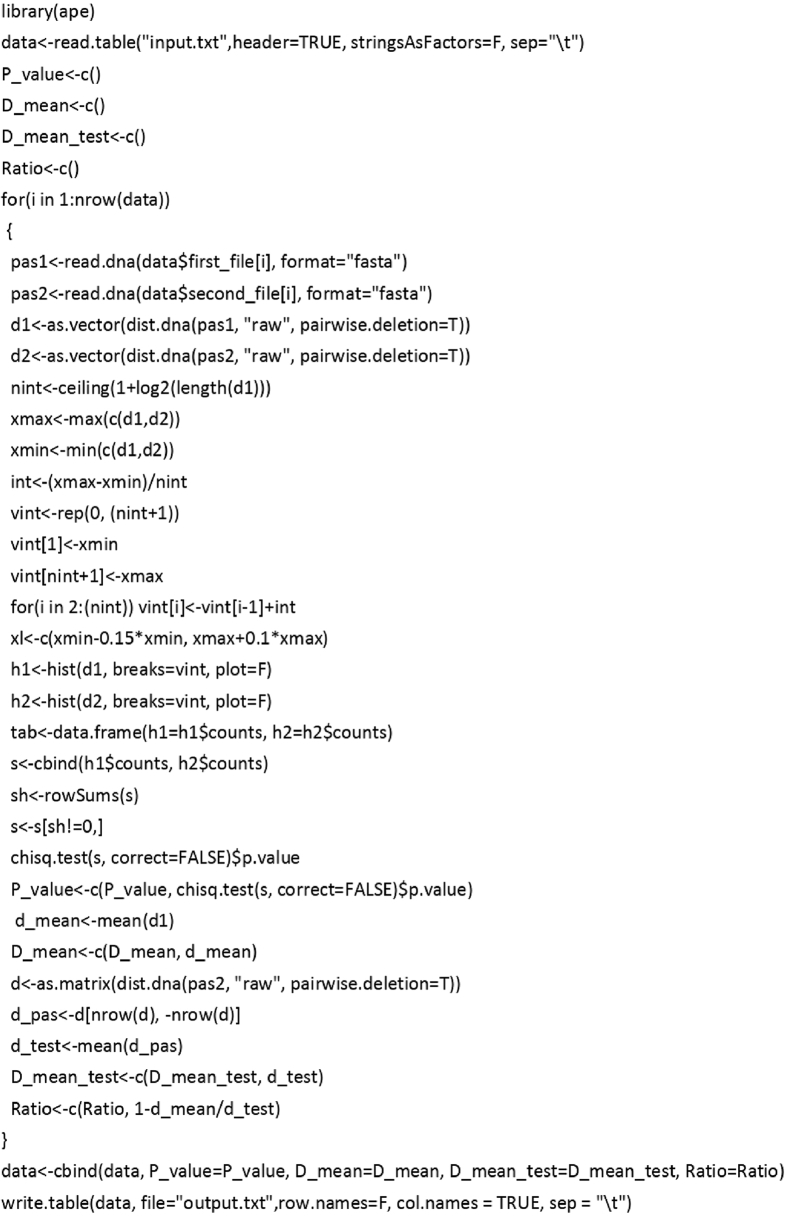

The distributions of pairwise genetic distances were obtained for tRNA genes of interest placed among each isoacceptor tRNA gene set. The content of amphipods species used to create isoacceptor tRNA gene sets for each test is shown in Ref. [1]. tRNA gene sets of every isoacceptor type consist of two input files: the first one contains aligned tRNA genes of a certain isoacceptor type (d1) and the second one has the tested tRNA gene added to the d1 set, and the sequence of the same species as that being examined is removed (d2). The input file (in the script input.txt) is a tab delimited table looks like this:

**Figure:**
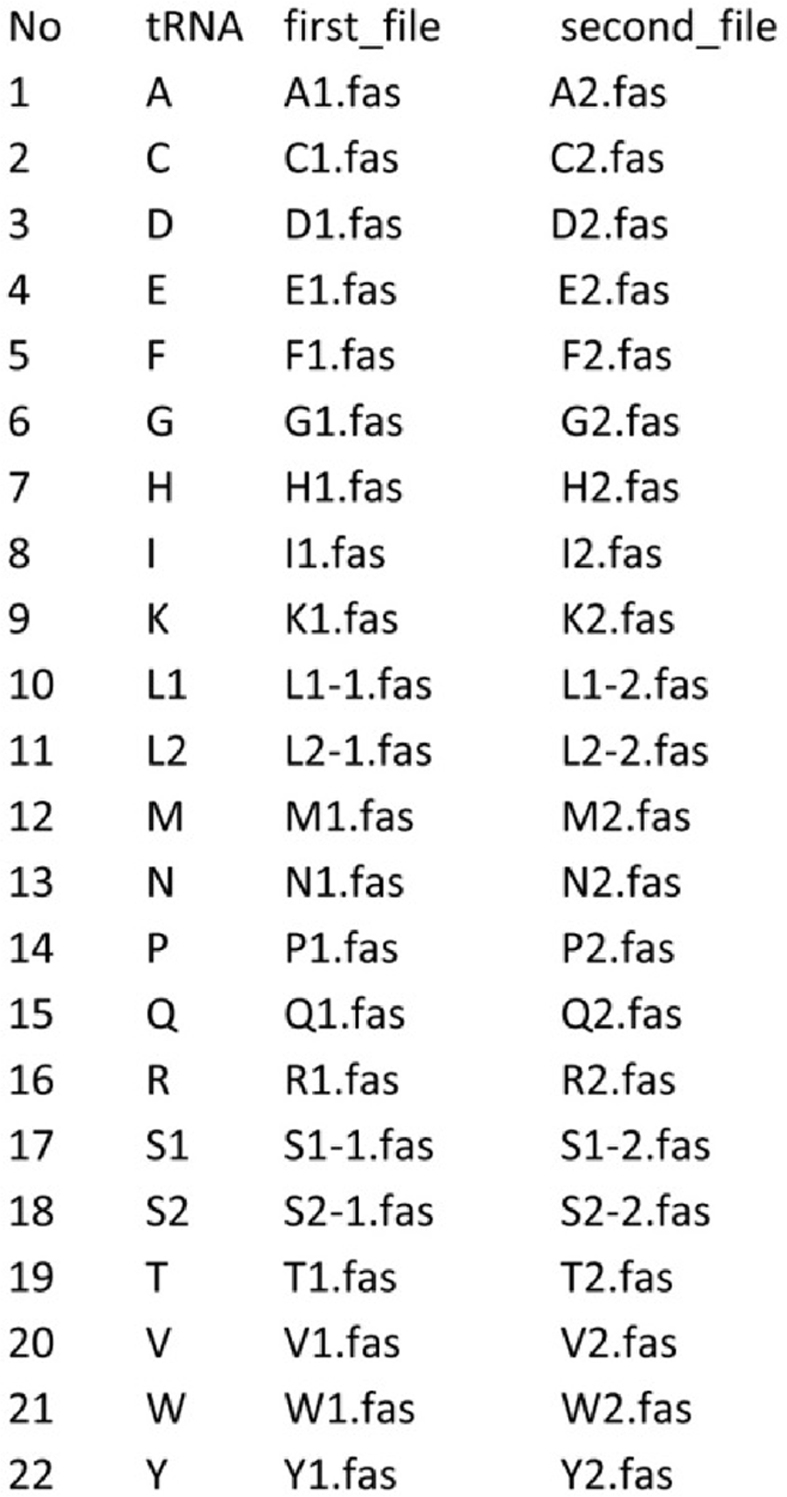

The first column is sequence numbers, the second column designates the tRNA gene isoacceptor type, the third column contains the names of aligned fasta files of d1 dataset for tRNA genes of every isoacceptor type (in the script from A1.fas to Y1.fas), and the fourth column contains the names of the aligned fasta files of d2 dataset for tRNA genes of every isoacceptor type (in the script from A2.fas to Y2.fas). The script calculates p-distances matrixes in d1 and d2 groups. These calculated values are used for building of histograms, ranging between the similar minimum and maximum values on the scale and subdivided into bins (number of bins is estimated using Sturges' formula [6]). The script then compares two histograms using the Fisher's chi-square test [7] and writes *p*-values in the fifths column of the output file (in the script output.txt):

**Figure:**
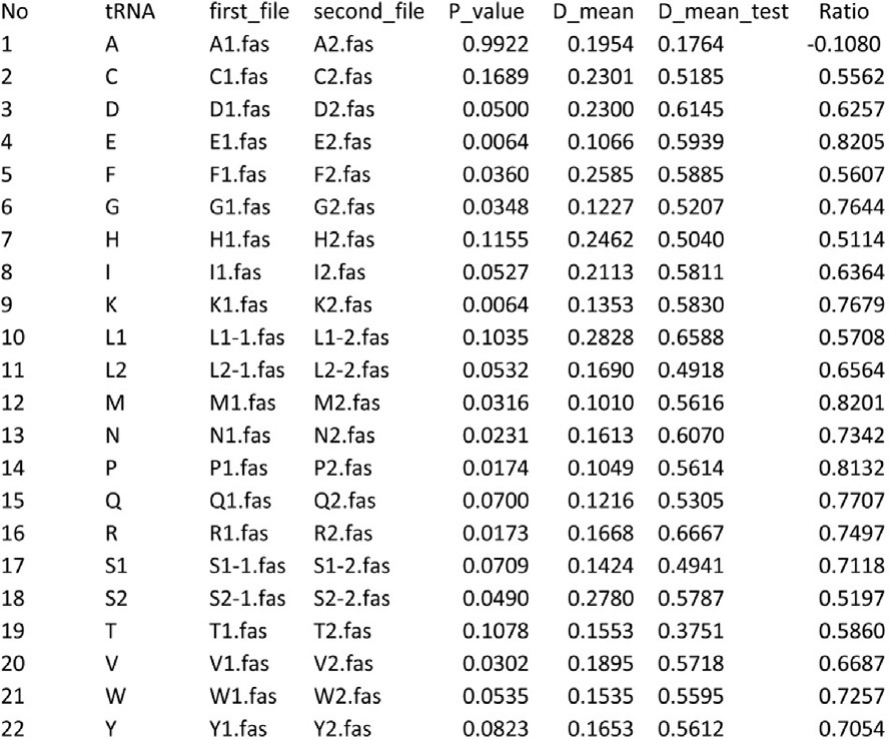

The script also calculates the mean distances (m1) for d1 groups and writes the values in the sixth column of the output file, and the mean distances (m2) between the sequence under examination has the latest position between the alignment and the rest of the sequences in d2, and the values are written in the seventh column of the output file. The ratio between m1 and m2 is calculated using formula 1-m1/m2, and the values are written in the eighth column of the output file. The maximum *p*-value and minimum value of the ratio between genetic distances indicate the type of the progenitor tRNA gene of the gene under study. For correct analysis, the mean p-distance of the aligned nucleotide sequences should not exceed 0.75.

### 2.2 R script for choosing the best sequence alignment

To perform phylogenetic analysis based on tRNA gene sequences, alignments considering different features of the secondary structures are often used [[8], [9], [10]]. The choice of the features is determined by the predicted structures of the tRNA molecules coded by the DNA fragments aligned. Numerous algorithms of the alignment of DNA fragments are also available. They perform differently in cases of short and hypervariable sequences such as tRNA genes. This causes the variation of topologies of trees inferred.

To determine the best mode of alignment among the several different alternatives, we developed an R script, which identifies the best alignment based on its minimum BIC value:

**Figure:**
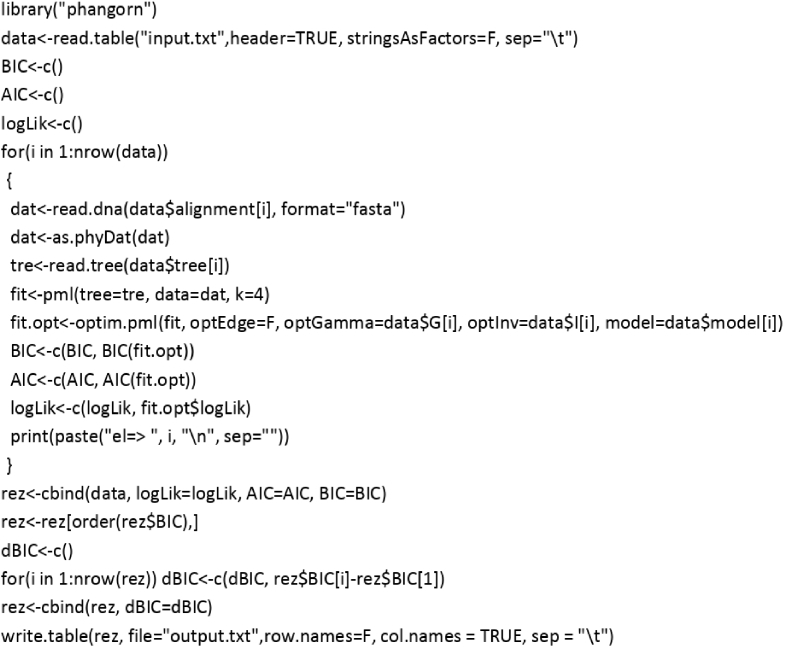

The dataset of every version of the alignment tested should contain an alignment file, a deduced substitution model, and the phylogenetic tree. The script utilizes the “phangorn” package [11]. The input file (in the script input.txt) is a tab delimited table looks like:

**Figure:**
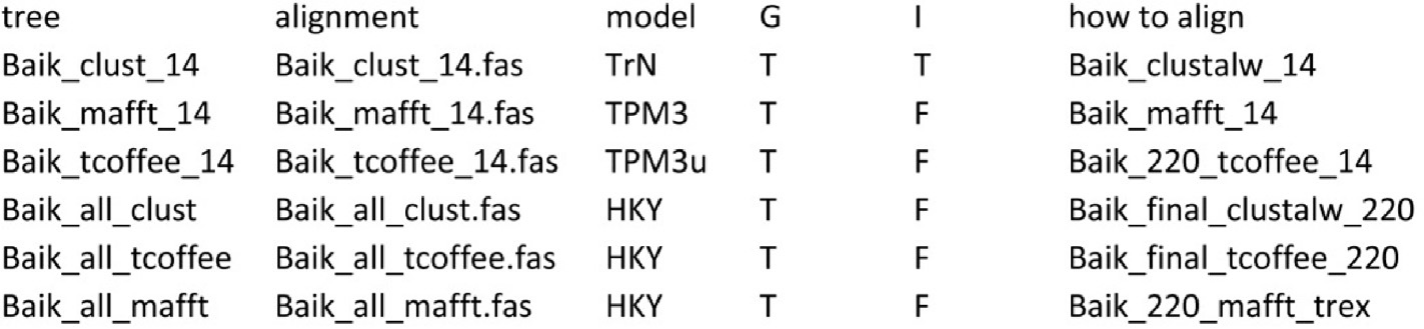

The first column contains names of tree files in Newick format, the second column contains names of aligned files in fasta format, the third column contains substitution model notation designated as in Posada (2008) [12], the forth column contains labels T or F (true of false) for the parameter of the gamma-shaped distribution of rates across sites that the model used, the fifth column contains labels T or F (true of false) for the proportion of invariable sites of the model used, and the sixth column contains any user's comments about alignments. The output file (in the script output.txt) is also a tab delimited table in which the columns with estimated mean likelihood, AIC, BIC, and delta BIC values for every alignment dataset are added:

**Figure:**
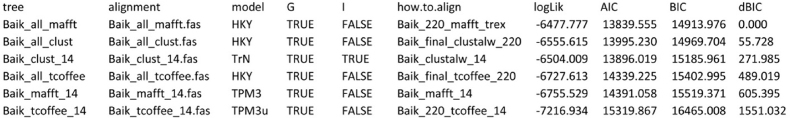

The dataset lines in the output file are sorted from the minimum delta BIC value at the top to the maximum value at the bottom. The current R script is suitable for assessment of the best alignment modes of any sets of aligned nucleotide sequences.

## CRediT authorship contribution statement

Elena V. Romanova: Investigation, Validation, Writing - original draft, Visualization. Yurij S. Bukin: Software, Formal analysis, Dmitry Yu. Sherbakov: Conceptualization, Methodology, Resources, Writing – review & editing.

## Appendix A Supplementary data

Multimedia component 1 **Table S1**. Pairwise identity analysis of tRNA genes of interest from mt genomes of amphipods. The maximum *p*-value and the minimum genetic distance ratio value indicate the isoacceptor type of the tested tRNA gene. **Table S2**. Comparison of different modes of tRNA gene alignment in different groups of amphipods. AIC is Akaike Information Criterion estimated for the current alignment mode. BIC is Bayesian Information Criterion estimated for the current alignment mode. Δ BIC is the difference between the BIC value of the current alignment mode and the BIC value of the best alignment mode (defined based on the minimum BIC value). Multimedia component 1
